# Let my father listen to my heart sounds too: Attachment in fathers who perform Leopold maneuvers and listen to fetal heart sounds, attachment and partner relationships in mothers

**DOI:** 10.1111/jjns.70002

**Published:** 2025-02-27

**Authors:** Esra Karataş Okyay, Esra Güney

**Affiliations:** ^1^ Department of Midwifery Kahramanmaraş Sütçü İmam University Kahramanmaraş Turkey; ^2^ Department of Midwifery İnönü University Malatya Turkey

**Keywords:** attachment, fetal heart sounds, Leopold maneuvers, midwifery, partner relationships

## Abstract

**Objective:**

This study was carried out to determine the effects of having expectant fathers perform the Leopold maneuvers and listen to fetal heart sounds on antenatal attachment in expecting couples and partner relationships in pregnant women.

**Materials and Methods:**

The sample of the experimental study consisted of 132 women, 132 men, constituting 66 couples in the experimental group and 66 in the control group. The expectant fathers in the experimental group performed the Leopold maneuvers. They listened to fetal heart sounds for 5–10 min at every practice. In both groups, the pregnant women filled out the Personal Information Form (PIF), the Maternal Antenatal Attachment Scale (MAAS), and the Prenatal Self‐Evaluation Questionnaire‐Relationship with Partner Subscale (PSEQ‐RPS), whereas the expectant fathers filled out the PIF and the Paternal Antenatal Attachment Scale (PAAS).

**Results:**

After the expectant fathers in the experimental group performed the Leopold maneuvers and listened to the heart sounds of their fetuses, the pregnant women in the experimental group had significantly higher total MAAS scores and significantly lower total PSEQ‐RPS scores than those in the control group (respectively, *p* < .001 and *p* < .05). The total PAAS scores of the expectant fathers in the experimental group were significantly higher than the total PAAS scores of those in the control group (*p* < .001).

**Conclusion:**

It was determined that having expectant fathers perform the Leopold maneuvers and listen to fetal heart sounds resulted in increased antenatal attachment levels in the expectant fathers and pregnant women and affected the relationships between the pregnant women and their partners positively.

## 1 INTRODUCTION

Pregnancy is a life event that entails substantial physiological and psychological adjustments for the mother. During pregnancy, some trimester‐specific adaptation processes are expected to develop (Ossa et al., 2012). One of these is that the mother starts feeling fetal movements at the end of the second trimester. The mother's perception of fetal movements is one of the first signs of fetal life and is considered an indicator of fetal well‐being (Flenady et al., 2019). From the 18th to the 25th weeks of gestation, the perception of fetal movements enables the mother to differentiate between herself and the fetus and consider the fetus an entity with needs, forms of communication, and intentionality (Cannella, 2005; Laxton‐Kane & Slade, 2002; Perricone et al., 2017). During this period, antenatal attachment starts to develop. Antenatal attachment is about the parent's affects, cognitions, and behaviors toward the fetus, such as name attribution, interaction with the fetus, speaking to the fetus, stroking the belly, prenatal fetal care, and physical preparation (Condon, 1993; Cranley, 1981; Muller, 1993). Having a baby is also a great evolutionary change for men who are experiencing fatherhood for the first time. However, the emotions of fathers have been taken into account less frequently in comparison to the psychological changes experienced by mothers (Bartlett, 2004). The transition to fatherhood roles is a complex process, just like the development of motherhood roles (Gulec & Kavlak, 2013). According to Condon (1985), paternal–fetal attachment is a subjective feeling of love for the unborn child, rather than an attitude or belief about the child, and it is at the heart of a man's experience of early parenting. This subjective love for the unborn child provides paternal attachment and thus the formation of the father's identity (Salehi et al., 2018). During this period, the mother plays a key role in the assumption of his paternal identity, mediating and supporting the father's experience (Condon et al., 2013).

The transition to parenthood is a major life event characterized by profound changes for a considerable number of new parents (George et al., 2013). Even though the arrival of a baby is often a joyful event, it can affect interpersonal communication. This transition is a transformative experience that changes the self‐concept, social roles, and daily routines of individuals (Saxbe et al., 2018). Therefore, it is associated with more stress and decreases the marital satisfaction of both partners (Mirowsky & Ross, 2002). The quality of the relationship of partners depends on their adaptation to this new status. A growing number of studies have looked at different characteristics of couples during the early period of their transition to parenthood, drawing some conclusions regarding the trajectory of marital satisfaction. Mitnick et al. (2009) found a small decrease in marital satisfaction during the first 11 postpartum months (Mitnick et al., 2009). Twenge et al. (2003) reported that following the first 2 years after the first child's birth, marital satisfaction substantially decreased (Twenge et al., 2003). Furthermore, a review that included 14 empirical studies on newlyweds concluded that a decrease in marital satisfaction was due to initially low levels of marital satisfaction or the inadequacy of essential experiences like the transition to parenthood (Proulx et al., 2017). Two perspectives have been identified regarding the fundamental nature of marital satisfaction during the transition to parenthood. While the first approach assumes that partners experience qualitative changes in their relationships, the other approach expresses the small, temporary, and quantitative character of decline in marital satisfaction (Lawrence et al., 2008). The attachment theory deepens the explanation of these perspectives (Bowlby, 1988). A partner's secure attachment has positive effects on marital satisfaction during the postpartum period through the high levels of support they provide and their optimistic expectations from their partner.

In the literature review, it was determined that prenatal attachment increased in mothers and fathers who listened to fetal heart sounds (Benli & Aksoy Derya, 2023), and the sharing that takes place between couples during pregnancy to prepare for childbirth (e.g., attending pregnancy follow‐ups, ultrasound scans together) had positive effects on the partner relationship (Drysdale et al., 2022; Harpel & Barras, 2017). It was reported that prenatal attachment would also increase in couples with a positive partner relationship (Kucukkaya et al., 2020). Building on the information in the relevant literature, we aim to investigate whether listening to fetal heartbeats is effective in increasing maternal‐paternal antenatal attachment and partner relationships. According to our primary outcome measure, based on the results of previous studies and to address the gap in the literature, we hypothesized that H_1a_:Paternal attachment increases in couples who perform the Leopold maneuvers and listen to fetal heart sounds. Considering our secondary outcomes, our hypotheses were H_1b_:Maternal attachment increases in couples who perform the Leopold maneuvers and listen to fetal heart sounds, and H_1c_:Partner relationships improve in couples who perform the Leopold maneuvers and listen to fetal heart sounds.

## 2 MATERIALS AND METHODS

This study was carried out with an experimental design involving a pretest, a posttest, and a control group. The study was conducted in the NST (nonst) outpatient clinics of a state hospital in a province in eastern Türkiye. The sample size required to conduct the study was calculated as 60 couples for either group, assuming a 3‐point increase in the mean score of paternal antenatal attachment (based on a mean of 64.5 with a standard deviation of 6.6 in a previous study) with a significance level of 5% in a one‐tailed test, an 80% power to represent the population, and a 95% confidence interval (Benli & Aksoy Derya, 2023). Considering a potential dropout rate of 10%, 66 couples were included in each group (66 couples in the experimental group and 66 in the control group) (Sakpal, 2010).

*Inclusion criteria for women*

- Being able to communicate,
- Being 18 years of age or older,
- Being in one's first legally recognized marriage,
- First‐time or previous mothers,
- Being at 28–36 weeks of gestation (the week intervals were determined based on the Ministry of Health Prenatal Care Management Guidelines. The third follow‐up week interval recommended in the guideline and the start date of the fourth follow‐up week were taken as a basis to ensure that the data collection process lasted for 2 weeks and avoid data loss) (Sağlık Bakanlığı Halk Sağlığı Genel Müdürlüğü, 2018),
- Having no diagnosed risks in the current pregnancy (e.g., preeclampsia, diabetes, heart disease, placenta previa, oligohydramnios, and multiple pregnancy),
- Having no diagnosed problems related to fetal health (e.g., fetal anomaly, intrauterine growth restriction).

*Inclusion criteria for men*

- Being able to communicate,
- Being 18 years of age or older,
- Being in one's first legally recognized marriage,
- First‐time or previous fathers,

*Exclusion criteria*

- Being separated from one's spouse,
- Pregnancy week in the first two trimesters,
- Couples with any identified risk to the fetus.

Among 71 couples who were initially included in the experimental group, one couple was excluded from the study because they moved out of the city while the study was going on, two were excluded because they said the process of listening to fetal heart sounds was tiring and did not want to continue the study, and two were excluded because they did not want to fill out the data collection forms for the second time. Among 68 couples who were initially included in the control group, two couples were excluded from the study because they did not want to fill out the posttest data collection forms. The study was completed with a total of 132 couples, 66 in the experimental group and 66 in the control group.

### 2.1 Randomization

**FIGURE 1:**
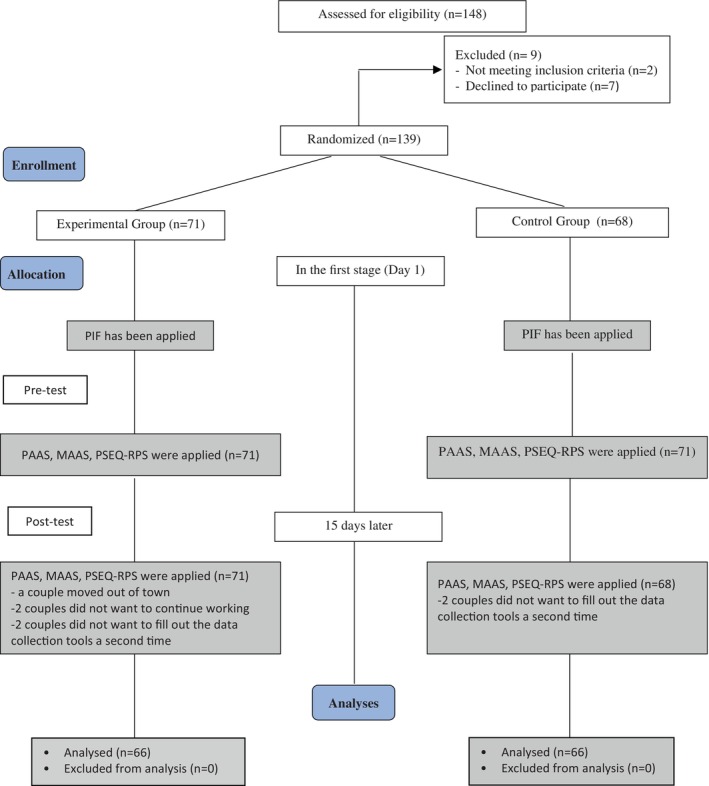
CONSORT diagram of participants for each stage in this study. MAAS, Maternal Antenatal Attachment Scale; PAAS, Paternal Antenatal Attachment Scale; PIF, Personal Information Form, PSEQ‐RPS, Prenatal Self‐Evaluation Questionnaire‐Relationship with Partner Subscale.

Couples who agreed to participate in the study and met the inclusion criteria were included in the experimental and control groups by the simple randomization method, and the sample selection process was carried out based on the CONSORT criteria. To randomly assign couples that met the inclusion criteria to the groups, columns numbered 1–132 were created at the beginning of the study using the Random Integer Generator tool under the Numbers page on the random.org website. A lot was drawn to randomly assign numbers to the groups. According to the result of the draw, the number 1 was assigned to the experimental group, while the number 2 was assigned to the control group. The randomization process was evaluated according to the criteria published by CONSORT to determine the quality of such studies and is shown in Figure 1.

### 2.2 Data collection

Data were collected in the NST outpatient clinics of a public hospital located in eastern Türkiye from December 1, 2023 to March 1, 2024 through face‐to‐face interviews using a questionnaire in two stages.

#### 2.2.1 Introduction, intervention, and pretest (day 1)

On the first day, couples who met the inclusion criteria were provided with information about the study. Written informed consent was obtained from those who agreed to participate. The pretest data collection phase was completed by applying data collection forms. Then, the researchers gave training to the expectant fathers in the experimental group using visual materials on how to perform the first and second Leopold maneuvers. At the beginning of the practice, how to perform the maneuvers was explained theoretically with materials prepared using colored pictures, and then, it was explained practically on the expectant mothers. In the first phase of the practical demonstration, the first and second Leopold maneuvers were applied by the researcher, and immediately afterward, the practices were carried out with the expectant father. Each couple was provided with a stethoscope to listen to fetal heartbeats, and they were instructed to listen to fetal heartbeats for 5–10 min together (using the same stethoscope alternately, first the father, then the mother), at least once a day, for 15 days, by performing the Leopold maneuvers. To record the number of fetal heartbeats they listened to, a fetal heartbeat recording chart prepared by the researchers was given to the couples. At the end of the first meeting, an appointment was made for the second stage (15 days later), and the first stage of the study was completed in approximately 10–15 min for each couple.

#### 2.2.2 Posttest (day 15)

The researchers interviewed the couples again according to the determined appointment date. During this meeting, the stethoscopes given to the couples so that they could listen to the heartbeats of their fetuses were collected. The fetal heart rate recording chart was checked, and the couples were informed that they could keep this chart if they wanted. Then, the data collection tools were applied again, and posttest data were collected. Hence, the second stage was completed in approximately 5–7 min for each couple.

No intervention was applied to the couples in the control group. Additionally, pretest (day 1) and posttest data (day 15) were collected by applying the data collection tools simultaneously with the experimental group. The couples in the control group were not informed about the intervention before the data collection phase was completed. However, on the day the data collection phase was completed (day 15—after the posttest data collection forms were applied), the couples in the control group were informed about the intervention carried out in the study. Charts and stethoscopes were provided by the researcher to 58 couples in the control group who wanted to count fetal heartbeats. Thus, equal participation rights were provided to all participants in the study.

### 2.3 Leopold maneuvers

Two maneuvers are the prerequisites for listening to a fetus' heart sounds. First, the mother's fundus is detected, and then, the baby's position is identified. Once the positioning shows which side of the mother's back the baby's back is on, fetal heart sounds can be easily listened to.

#### 2.3.1 Maneuver 1 (fundal grip)

The first maneuver is performed to determine the height of the fundus and the part located in the fundus. The person performing the maneuver faces the pregnant woman. The practitioner moves their hands up the side of the uterus, gently palpating the fundus. If the part located in the fundus is the anus, it gives the feeling of a soft mass with less sharp borders and not round. If it is the head, it gives the impression of a solid, round, and mobile mass with regular borders.

#### 2.3.2 Maneuver 2 (lateral or umbilical grip)

This maneuver is applied to determine which side the fetus' back is on. The person performing the maneuver faces the pregnant woman. The palms of both hands are placed on the abdomen on the right and left sides of the uterus. An attempt is made to identify which side the back is on by keeping the hand on one side stationary and palpating the uterus with the fingertips of the other hand. During palpation, the side where the baby's back is located is felt to be flatter and fuller, while the other side is felt to be more indented and protruding.

### 2.4 Data collection tools

Data were collected using a Personal Information Form (PIF), the Paternal Antenatal Attachment Scale (PAAS), the Maternal Antenatal Attachment Scale (MAAS), and the Prenatal Self‐Evaluation Questionnaire‐Relationship with Partner Subscale (PSEQ‐RPS).

#### 2.4.1 Personal Information Form

This form included questions to determine the sociodemographic characteristics of the couples (e.g., age, education level, employment status, income level) and the obstetric characteristics of the expectant mothers (e.g., number of pregnancies, gestational age, obstetric history).

#### 2.4.2 Paternal Antenatal Attachment Scale

The scale developed by Condon (1993) consists of 16 items and is a 5‐point Likert‐type scale. Each item of the scale focuses on measuring the feelings, attitudes, behaviors, and thoughts of the father toward the developing fetus in the womb. Most of the items are based on the experiences of the expectant father over the past 2 weeks. The Turkish validity and reliability study of the scale was conducted by Benli and Aksoy Derya (2021). The scale has two dimensions: “quality of attachment (eight items)” and “intensity of preoccupation (eight items).” Each item has a score range of 1–5, and the total score range of the scale is 16–80. Cronbach's alpha reliability coefficient for the scale was reported as .82, and the scale has no cutoff point. Higher scores are accepted to indicate higher prenatal attachment. In this study, the Cronbach's alpha coefficient of the scale was found to be .75.

#### 2.4.3 Maternal Antenatal Attachment Scale

The scale was developed by Condon (1993), and its validity and reliability study in Turkish was conducted by Golbasi et al. (2015). All items of the scale, consisting of a total of 19 items, focus on the feelings, attitudes, and behaviors of pregnant women toward their fetuses. A 5‐point Likert‐type scoring system is used for each item, with item scores ranging from 1 to 5 (5 represents strong emotions toward the fetus, whereas 1 represents the absence of feelings toward the fetus). The scale has two dimensions. The quality of attachment dimension represents the quality of the emotional experiences of a pregnant woman regarding the fetus. The intensity of preoccupation dimension represents the intensity of the pregnant woman's preoccupation with the fetus and thinking about the fetus, talking with it, and touching it through her belly. High scores indicate high levels of attachment. In this study, the Cronbach's alpha coefficient of the scale was found to be .79.

#### 2.4.4 Prenatal Self‐Evaluation Questionnaire‐Relationship with Partner Subscale

PSEQ was developed by Lederman et al. (2009) to identify women's adaptation to pregnancy and motherhood. The validity and reliability study of the scale form in Turkish was carried out by Beydağ and Mete (2008) (Beydağ & Mete, 2008). PSEQ is a 4‐point Likert‐type scale with seven subscales and 79 items. In this study, the Relationship with Partner Subscale (PSEQ‐RPS) was used. The relationship with partner dimension of the scale consists of 10 items (items 4, 5, 10, 23, 35, 36, 40, 43, 60, and 70). The items in the dimension are as follows: “my partner and I talk about our unborn baby,” “my partner has criticized me throughout my pregnancy,” “my partner shows me compassion when I am sad,” “my partner likes talking about my pregnancy with me,” “my partner helps household chores when I need help,” “I find it difficult to talk to my partner about changes in our sex life during my pregnancy,” “I believe my partner will support me during childbirth,” “my partner thinks I bore him with my emotions and problems,” “I believe my partner can talk to me about our sex life during my pregnancy,” and “I trust my partner in sharing the care responsibility of our baby.” The score range of the 10‐item PSEQ‐RPS is 10–40. High scores indicate poor adaptation, whereas low scores indicate increased adaptation. The Cronbach's alpha coefficient of PSEQ‐RPS was reported as .78 (Beydağ & Mete, 2008). In this study, the Cronbach's alpha coefficient of the scale was found to be .72.

### 2.5 Outcomes

The primary outcome of this study was the attachment levels of fathers during the antenatal period (PAAS). The secondary outcomes were the attachment levels of mothers (MAAS) and their partner relationships (PSEQ‐RPS).

### 2.6 Statistical analysis

The data were analyzed using the SPSS 25.0 for Windows software (SPSS, Chicago, IL, USA). The data were visualized with the R software programming language, and descriptive statistics were calculated as frequencies, percentages, means, and standard deviations. Independent samples *t*‐tests were used for comparisons between the experimental and control groups, and paired samples *t*‐tests were used for intragroup comparisons. To test the effects of the intervention, an analysis of covariances (ANCOVA) was performed to check the pretest scores, parity, and gestational week (start‐day 1) of the participants. Because the baseline attachment and partner relationship levels of the participants could affect their levels in the posttest, their pretest scores were taken as covariates. Parity and gestational week (start‐day 1) were also taken as covariates in the ANCOVA to control for their effects on the dependent variables between the groups. The effect sizes are reported as partial *η* ^2^ (eta‐squared) values. According to the information in the literature, effect sizes are categorized as “small” when 0.01 ≤ *η* ^2^ < 0.06, “medium” when 0.06 ≤ *η* ^2^ < 0.14, and “large” when *η* ^2^ ≥ 0.14 (Cohen, 1988). *p* < .05 was considered statistically significant.

### 2.7 Ethical considerations

For conducting the study, ethical approval was obtained from the Inonu University Noninvasive Clinical Research and Publication Ethics Committee (Decision No: 2023/4161), permission was received from the institution where the data would be collected, and the protocol of the study was registered at www.clinicaltrials.gov (NCT06148415). All couples in the study were informed about the study on the first page of the data collection tools, and their informed consent was obtained.

## 3 RESULTS

There was no statistically significant difference between the experimental and control groups in terms of their sociodemographic or obstetric characteristics (*p* > .05; Table 1).

**TABLE 1:** Descriptive characteristics of the participants (*n* = 132 women, *n* = 132 men).

Descriptive characteristics	Experimental group (*n* = 66)	Control group (*n* = 66)	Test	*p* Values
	*M* ± SD	*M* ± SD		
Age (years)^a^	28.55 ± 3.91	29.27 ± 4.67	*t* = 0.969	*p =* .334^b^
Gestational week (start‐day 1)^a^	34.24 ± 1.43	34.50 ± 2.08	*t* = 0.827	*p =* .410^b^
Gestational week (end‐day 15)^a^	36.24 ± 1.43	36.50 ± 2.08	*t* = 0.827	*p =* .410^b^
Duration of marriage (years)^a^	5.54 ± 4.39	6.24 ± 4.60	*t* = 0.899	*p =* .370^b^
Spouse's age (years)^c^	31.88 ± 5.23	32.45 ± 6.32	*t* = 0.570	*p =* .570^b^

Descriptive characteristics	Experimental group (*n* = 66)		Control group (*n* = 66)		Test	*p* Values
	*n*	%	*n*	%		
Educational level^a^						
≤12 year	45	68.2	39	59.1	*χ* ^2^ = 0.818	*p =* .366^d^
>12 year	21	31.8	27	40.9		
Spouse's educational level^c^						
≤12 year	28	42.4	23	34.8	*χ* ^2^ = 0.799	*p =* .371^e^
>12 year	38	57.6	43	65.2		
Social security^a^						
Yes	58	87.9	56	84.8	*χ* ^2^ = 0.064	*p =* .800^d^
No	8	12.1	10	15.2		
Employment status^a^						
Yes	24	36.4	14	21.2	*χ* ^2^ = 2.993	*p =* .084^d^
No	42	63.6	52	78.8		
Spouse's employment status^c^						
Yes	62	93.9	57	86.4	*χ* ^2^ = 1.365	*p =* .243^d^
No	4	6.1	9	13.6		
Perceived income level^a^						
Income is less than expenses	12	18.2	10	15.2	*χ* ^2^ = 0.222	*p =* .895^e^
Income is equal to expenses	49	74.2	51	77.3		
Income is more than expenses	5	7.6	5	7.5		
Family type^a^						
Nuclear family	56	84.8	54	81.8	*χ* ^2^ = 0.055	*p =* .815^d^
Extended family	10	15.2	12	18.2		
Parity^a^						
Primigravida	31	47.0	41	62.1	*χ* ^2^ = 3.056	*p =* .080^e^
Multigravida	35	53.0	25	37.9		
Abortion history^a^						
Yes	21	31.8	18	27.3	*χ* ^2^ = 0.146	*p =* .703^d^
No	45	68.2	48	72.7		
The status of having a planned pregnancy^a^						
Planned	54	81.8	46	69.7	*χ* ^2^ = 2.021	*p =* .155^d^
Unplanned	12	18.2	20	30.3		
Form of marriage^a^						
Arranged marriage	20	30.3	26	39.4	*χ* ^2^ = 0.834	*p =* .361^d^
By agreement/willingly	46	69.7	40	60.6		

^a^ Descriptive characteristics of women.

^b^ İndependent samples *t*‐test.

^c^ Descriptive characteristics of men.

^d^ Continuity Correction.

^e^ Pearson chi‐squared test, *M* (mean), SD (standard deviation).

**FIGURE 2:**
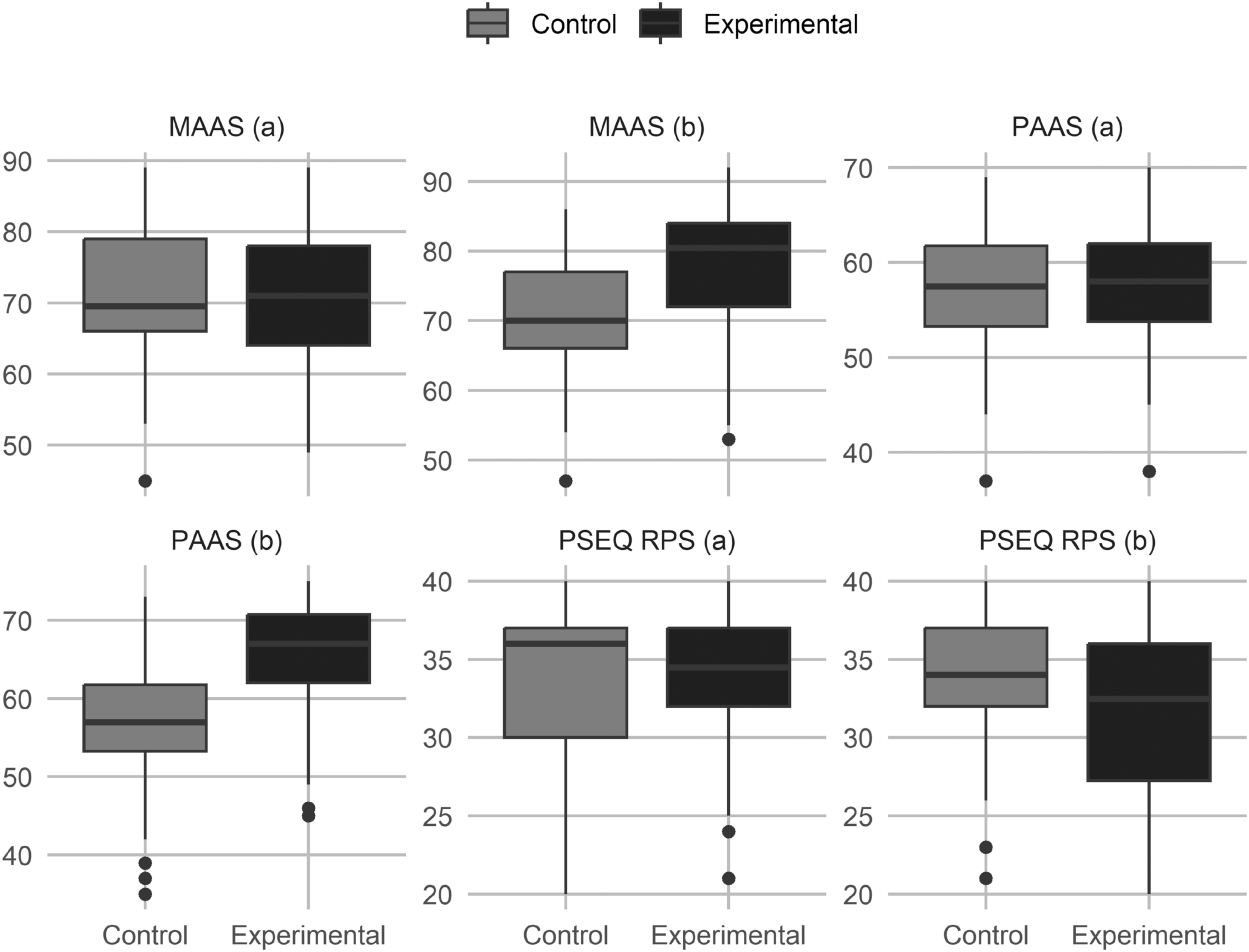
Mean Maternal Antenatal Attachment Scale (MAAS), Prenatal Self‐Evaluation Questionnaire‐Relationship with Partner Subscale (PSEQ‐RPS), and Paternal Antenatal Attachment Scale (PAAS) scores of the participants. (a): Pretest, (b): Posttest.

It was determined that in the pretest, there was no significant difference between the women in the experimental group and those in the control group in terms of their MAAS total scores, MAAS subscale scores, or PSEQ‐RPS scores (*p* > .05), whereas the score differences between the experimental and control groups in the posttest were statistically significant in favor of the experimental group (MAAS total and MAAS Quality of Attachment subscale scores: *p* < .001, others: *p* < .05). In the intergroup comparisons of the changes in the scores of the women from the pretest to the posttest, it was observed that the MAAS total and subscale scores of the women in the experimental group increased significantly (MAAS Quality of Attachment subscale: *p* < .05, others: *p* < .001), while their PSEQ‐RPS scores significantly decreased (*p* < .05) (Table 2; Figure 2).

**TABLE 2:** Intragroup and intergroup comparisons of the pretest‐posttest Maternal Antenatal Attachment Scale (MAAS) and Prenatal Self‐Evaluation Questionnaire‐Relationship with Partner Subscale (PSEQ‐RPS) scores of women.

Scales	Measurements	Experimental group (*n* = 66), *M* ± SD	Control group (*n* = 66), *M* ± SD	Test^a^	*p* Values
MAAS	Attachment quality				
	Pretest	39.27 ± 4.82	38.56 ± 5.12	*t* = −0.801	*p =* .413
	Posttest	41.92 ± 5.64	38.19 ± 5.12	*t* = −3.972	***p* < .001** *
	Test^b^ and *p* value	*t* = −3.449 ***p =* .001**	*t* = 0.525 *p =* .601		
	Time spent on attachment				
	Pretest	27.37 ± 4.54	28.24 ± 4.95	*t* = 1.043	*p =* .299
	Posttest	31.09 ± 4.72	29.04 ± 4.12	*t* = −2.650	***p =* .009**
	Test^b^ and *p* value	*t* = −5.641 ***p* < .001** *	*t* = −1.453 *p =* .151		
	MAAS total				
	Pretest	70.25 ± 8.96	70.89 ± 8.80	*t* = 0.412	*p = .681*
	Posttest	77.33 ± 10.21	71.34 ± 7.90	*t* = −3.765	***p* < .001** *
	Test^b^ and *p* value	*t* = −5.099 ***p* < .001** *	*t* = −0.450 *p =* .654		
PSEQ‐RPS	Pretest	34.22 ± 3.88	33.72 ± 5.53	*t* = −0.601	*p = .549*
	Posttest	31.68 ± 6.08	33.71 ± 4.34	*t* = 2.204	***p =* .029**
	Test^b^ and *p* value	*t* = 3.002 ***p =* .004**	*t* = 0.018 *p =* .985		

*Note*: Italics indicate significant values. Bold indicates significant values.

Abbreviations: *M*, mean; SD, standard deviation.

^a^ İndependent samples *t*‐test.

^b^ Paired‐samples *t*‐test.

* *p* = .000.

The pretest PAAS total and subscale scores of the men in the experimental group and those in the control group were not significantly different from each other (*p* > .05), whereas the groups differed significantly in terms of their posttest scores in favor of the experimental group (all: *p* < .001). In the intragroup comparisons, it was seen that the PAAS total and subscale scores of the men in the experimental group increased significantly after the intervention (all: *p* < .001) (Table 3; Figure 2).

**TABLE 3:** Intragroup and intergroup comparisons of the pretest‐posttest Paternal Antenatal Attachment Scale (PAAS) scores of men.

Scale	Measurements	Experimental group (*n* = 66), *M* ± SD	Control group (*n* = 66), *M* ± SD	Test^a^	*p* Values
PAAS	Quality of attachment				
	Pretest	31.04 ± 4.13	30.81 ± 4.59	*t* = −0.299	*p = .766*
	Posttest	34.78 ± 4.85	31.43 ± 5.46	*t* = −3.722	***p* < .001** *
	Test^b^ and *p* value	*t* = −5.723 ***p* < .001** *	*t* = −0.869 *p =* .388		
	Time spent in attachment mode				
	Pretest	26.66 ± 3.66	26.31 ± 4.35	*t* = −0.497	*p = .620*
	Posttest	30.13 ± 3.33	25.36 ± 4.52	*t* = −6.892	***p* < .001** *
	Test^b^ and *p* value	*t* = −5.914 ***p* < .001** *	*t* = 1.661 *p =* .102		
	PAAS total				
	Pretest	57.71 ± 6.66	57.13 ± 6.90	*t* = −0.487	*p = .627*
	Posttest	64.92 ± 7.27	56.80 ± 7.76	*t* = −6.200	***p* < .001** *
	Test^b^ and *p* value	*t* = −6.205 ***p* < .001** *	*t* = 0.343 *p =* .732		

*Note*: Bold indicates significant values.

Abbreviations: *M*, mean; SD, standard deviation.

^a^ İndependent samples *t*‐test.

^b^ Paired‐samples *t*‐test.

* *p* = .000.

According to the results of the ANCOVA, when the pretest MAAS total scores, PSEQ‐RPS scores, PAAS scores, parity values, and gestational weeks (start‐day 1) of the participants were controlled for, a statistically significant difference was identified between the posttest scores of the participants in the experimental group and those in the control group (respectively, *F*: 17.635, *p* < .001; *F*: 4.355, *p*: .039; *F*: 36.590, *p* < .001). This difference was in favor of the men and women in the experimental group (MAAS ${\bar{X}}_{\text{experiment}}$: 77.33, ${\bar{X}}_{\text{control}}$: 71.34; PSEQ‐RPS ${\bar{X}}_{\text{experiment}}$: 31.68, ${\bar{X}}_{\text{control}}$: 33.71; PAAS ${\bar{X}}_{\text{experiment}}$: 64.92, ${\bar{X}}_{\text{control}}$: 56.80). After controlling for pretest scores and variable values, this result showed that the performance of the Leopold maneuvers by the men and having men listen to fetal heart sounds resulted in a significant increase in the antenatal maternal–fetal attachment levels and a significant improvement in the partner relationships of the women and a significant increase in the antenatal paternal–fetal attachment levels of the men. As a metric of effectiveness, the partial *η* ^2^ values of the outcome measures were found to be 0.122 for MAAS, 0.033 for PSEQ‐RPS, and 0.224 for PAAS. Neglecting the effect of the covariate, it was concluded that the intervention was 12.2% effective (medium effect size) on the antenatal maternal–fetal attachment levels of the women, 3.3% effective (small effect size) on the partner relationships of the women, and 22.4% effective (large effect size) on the antenatal paternal–fetal attachment levels of the men (Table 4).

**TABLE 4:** Analysis of covariances results of the corrected posttest Maternal Antenatal Attachment Scale (MAAS) total, Prenatal Self‐Evaluation Questionnaire‐Relationship with Partner Subscale (PSEQ‐RPS), and Paternal Antenatal Attachment Scale (PAAS) total scores of the participants (*n* = 132 women, *n* = 132 men).

Scales	Resources	Sum of squares	df	Average of squares	*F*	*p*	*η* ^2^
MAAS total	Pretest	1663.184	1	1663.184	23.369	<.001	0.155
	Parity	6.597	1	6.597	0.093	.761	0.001
	Gestational week (start‐day 1)	46.473	1	46.473	0.653	.421	0.005
	Group	**1255.115**	**1**	**1255.115**	**17.635**	**<.001** *	**0.122**
	Error	9038.776	127	71.171			
	Total	741531.000	132				
	Experimental group [posttest *M* (SD)–AM *M* (SE)]	77.33 (10.21)–77.47 (1.04)					
	Control group [posttest *M* (SD)–AM *M* (SE)]	71.34 (7.90)–71.21 (1.04)					
PSEQ‐RPS	Pretest	34.697	1	34.697	1.228	.270	0.010
	Parity	14.348	1	14.348	0.508	.477	0.004
	Gestational week (start‐day 1)	2.577	1	2.577	0.091	.763	0.001
	Group	**123.100**	**1**	**123.100**	**4.355**	.**039** **	**0.033**
	Error	3589.640	127	28.265			
	Total	144896.000	132				
	Experimental group [posttest *M* (SD)–AM *M* (SE)]	31.68 (6.08)–31.71 (0.66)					
	Control group [posttest *M* (SD)–AM *M* (SE)]	33.71 (4.34)–33.67 (0.66)					
PAAS total	Pretest	537.473	1	537.473	10.068	.002	0.073
	Parity	45.547	1	45.547	0.853	.357	0.007
	Gestational week (start‐day 1)	18.492	1	18.492	0.346	.557	0.003
	Group	**1953.317**	**1**	**1953.317**	**36.590**	**<.001** *	**0.224**
	Error	6779.719	127	53.384			
	Total	498516.000	132				
	Experimental group [posttest *M* (SD)–AM *M* (SE)]	64.92 (7.27)–64.77 (0.90)					
	Control group [posttest *M* (SD)–AM *M* (SE)]	56.80 (7.76)–56.95 (0.90)					

*Note*: Bold indicates significant values.

Abbreviations: *η* ^2^, partial eta squared; AM, adjusted mean; df, degrees of freedom; *F*, ANCOVA test with adjusting the baseline score, parity, and gestational week (start‐day 1) were used; *M*, mean; SD, standard deviation; SE, standard error.

* *p* = .000, **p < .05.

## 4 DISCUSSION

This study was carried out to determine the effects of having expectant fathers perform the Leopold maneuvers and listen to fetal heart sounds on antenatal attachment in expecting couples and partner relationships in pregnant women. It was found that having the expectant fathers perform the Leopold maneuvers and listen to the heart sounds of their unborn babies increased the antenatal attachment levels of both the expectant fathers and the pregnant women and improved the partner relationships of the pregnant women. The sociodemographic and obstetric characteristics of the participants in the experimental and control groups were similar, which showed that the participants were homogeneously distributed into the groups. In the pretest, the MAAS total and subscale, PSEQ‐RPS, and PAAS total and subscale scores of the participants did not differ significantly between the experimental and control groups. Therefore, before the intervention, the participants in the experimental and control groups had similar antenatal attachment levels, and the pregnant women in the experimental and control groups had similar partner relationship characteristics. This reinforced our confidence in our finding that the results obtained after the intervention originated from the intervention and not from the characteristics of the participants in the two groups.

The intervention that was performed in this study increased the antenatal maternal–fetal attachment levels of the pregnant women. The literature review that was conducted for this study did not reveal any previous study on the effects of having the expectant father perform the Leopold maneuvers and listen to fetal heart sounds on antenatal maternal–fetal attachment. In a study investigating the effects of self‐performed fetal movement and position monitoring on antenatal attachment and pregnancy‐related distress, it was seen that the intervention resulted in favorable outcomes in terms of antenatal attachment (Badem & Mucuk, 2022). A systematic review revealed that maternal–fetal attachment levels in pregnant women were raised by counting fetal movements (AlAmri & Smith, 2022), and similarly, the independent observation of fetal movements using audiovisual media was seen to increase the antenatal attachment levels of pregnant women (Purwati & Sari, 2024). In the studies conducted by Celik and Ergin (2020) and Nishikawa and Sakakibara (2013), who subjected pregnant women to the Leopold maneuvers, significant increases were observed in the antenatal attachment levels of pregnant women. In this study, it was aimed to actively involve the partners of pregnant women in the process of increasing antenatal parental–fetal attachment. The results reported by Setodeh et al. (2018) supported the result of our study and showed that attachment‐related education provided to expectant fathers increased maternal–neonatal attachment. In a study on antenatal paternal– and maternal–fetal attachment, it was determined that pregnant women who saw their fetuses in ultrasound with their partners had significantly higher antenatal attachment levels than those who saw their fetuses in ultrasound in the absence of their partners (Ugurlu et al., 2023). These findings supported our findings. Behaviors such as communicating with one's unborn baby and trying to locate the extremities and position of the baby by touching one's belly are seen as indicators of increased emotional connection between the pregnant woman and her fetus (Nishikawa & Sakakibara, 2013). Accordingly, the expectant father feeling the position of the fetus and listening to the heartbeats of his unborn baby may have increased the antenatal attachment levels of the mother as a consequence of sharing these moments with her partner.

It was reported that the involvement of spouses in the antenatal care and follow‐up process improved the partner relationships of couples (Drysdale et al., 2022; Kroelinger & Oths, 2000). In our study, while the relationships between the pregnant women and their spouses did not significantly change in the control group, the pregnant women in the experimental group had significantly improved partner relationships after the intervention. To the best of our knowledge, there is no study in the literature examining the effects of similar interventions that actively involve expectant fathers on partner relationship parameters. In a study investigating partner relationships during pregnancy, the involvement of spouses in antenatal care was identified as a significant factor that improved the partner relationships of women (Akkaş & Balkaya, 2014). Similar results were obtained in a study that was performed to identify the ultrasound scan experiences of partners, and it was found that participating in ultrasound examinations had positive effects on most men, and their relationships with their pregnant partners improved accordingly (Drysdale et al., 2022). In another study, it was stated that the participation of spouses in ultrasound examinations was an instrument for strengthening the support systems of pregnant women (Harpel & Barras, 2017). The results of other studies in the literature supported the results of this study.

In our study, having the expectant father perform the Leopold maneuvers and listen to the fetal heart sounds of his unborn baby resulted in an increase in his antenatal paternal–fetal attachment levels. According to the results of the literature review, no study has yet investigated the effects of having expectant fathers perform the Leopold maneuvers and listen to fetal heart sounds on antenatal paternal–fetal attachment. It was found that having couples listen to the heart sounds of their unborn babies increased antenatal paternal–fetal attachment (Benli & Aksoy Derya, 2023), while in another study conducted with a cross‐sectional design, higher levels of paternal–fetal attachment were reported in fathers who attended pregnancy follow‐ups with their spouses (Guler Kaya & Ayar Kocaturk, 2020). In their qualitative study, de Waal et al. (2023) reported that feeling the movements of the fetus facilitated paternal–fetal attachment in some fathers, and the intensity of these feelings increased as the fetus became more active. These results were compatible with those obtained in our study. Considering the results of both this study and other studies in the literature, it is believed that having the expectant father perform the Leopold maneuvers and listen to fetal heart sounds increases antenatal paternal–fetal attachment by facilitating the faster adaptation of the man to fatherhood roles and increasing his feelings of responsibility toward his unborn child.

### 4.1 Limitations and strengths

Although fathers have significant roles in all areas of life, especially during the pregnancy period, there are very few studies investigating this topic in Türkiye. Additionally, no study examining the effects of having the expectant father perform the Leopold maneuvers and listen to the heart sounds of his unborn child on antenatal paternal–maternal attachment and partner relationships was encountered in the literature. This was a strong aspect of our study. Another strength of this study was that expectant fathers were involved in the pregnancy process, and this situation affected the connection between the parents and the fetus positively. Furthermore, the involvement of expectant fathers in the pregnancy process also affected the partner relationships with their spouses positively. This can, in turn, increase the quality of the relationship between partners and help parents feel competent. Controlling for the pretest results, which could have influenced paternal–maternal attachment and partner relationships, in our study increased our confidence in the finding that the results of the study originated from the intervention. The need for manpower to teach the Leopold maneuvers and the need for stethoscopes to listen to fetal heart sounds can be considered weaknesses of the initiative.

One of the most important limitations of this study was the limited number of fathers attending follow‐ups with their spouses, as well as difficulties in involving male parents in the pregnancy process. The scales used in the study are subjective measurement instruments, and the results were based on the self‐reports of the participants. Collecting information through self‐reports brings about some issues. Individuals may be biased when they report information about themselves. Therefore, they may prefer to give more socially acceptable answers instead of answering the question truthfully. They may also not be able to assess themselves accurately. The way the questions are worded may also confuse participants. Questions may mean different things to different individuals. Additionally, answering something as yes or no may be limiting for individuals. Participants may tend to give an extreme or moderate response to all questions. Finally, questions may be influenced by answers given previously, which may create some bias (McDonald, 2008; Paulhus & Vazire, 2007; Salters‐Pedneault, 2023). For this reason, it is recommended that future studies be conducted with more participants and using objective measurement instruments. Another important limitation of our study was the inability to control the effect of factors such as skin‐to‐skin contact and speech that enable couples to interact during the intervention. One limitation was the fact that it was difficult to follow up on the completion of the fetal movement chart by the participants and reach them for the collection of posttest data. As the study had two stages, it was difficult to reach more participants. The inclusion of participants from only one center in the sample was also a limitation. Therefore, the results of this study may not be generalizable to the entire population, but they can be used for comparisons to the results of other studies. Additionally, as it was planned to evaluate only maternal‐paternal outputs during pregnancy in the designing stage of the study, the long‐term effects of the intervention were not studied. Researchers are recommended to investigate these effects by planning long‐term interventions and monitoring procedures. It should be noted as another limitation that factors other than the variables examined here that could have affected antenatal paternal–maternal attachment and partner relationships were not controlled in our study.

## 5 CONCLUSION AND RECOMMENDATIONS

Based on the results of this study, having the expectant father perform the Leopold maneuvers and listen to the heart sounds of his unborn baby increased the antenatal parental–fetal attachment levels of both parents‐to‐be. This intervention also improved the partner relationships of the pregnant women. Further studies are needed to confirm the findings of this study and discover the effects of similar interventions. Although the literature on this particular topic is limited, some studies conducted to increase parental attachment like our study have shown that interventions where expectant fathers take active roles in the antenatal period can help increase antenatal parental attachment and improve partner relationships. This is why it is important to encourage pregnant women to attend outpatient clinics with their partners and provide information, education, and counseling to both prospective parents. Considering all these results, in terms of observing the positive effects of having expectant fathers perform the Leopold maneuvers and listen to fetal heart sounds as an intervention that does not require any invasive procedure, is cost‐free, and is easy to perform, it is recommended that this practice be integrated into routine antenatal care.

## AUTHOR CONTRIBUTIONS

**Esra Karataş Okyay:** Conceptualization, methodology, formal analysis, writing—original draft, writing—reviewing and editing. **Esra Güney:** Conceptualization, methodology, data curation, formal analysis, writing—reviewing and editing.

## CONFLICT OF INTEREST STATEMENT

The authors declare that there is no conflict of interest.
